# A multidimensional approach to frailty compared with physical phenotype in older Brazilian adults: data from the FIBRA-BR study

**DOI:** 10.1186/s12877-021-02193-y

**Published:** 2021-04-14

**Authors:** Claudia Venturini, Rosana Ferreira Sampaio, Bruno de Souza Moreira, Eduardo Ferriolli, Anita Liberalesso Neri, Roberto Alves Lourenço, Lygia Paccini Lustosa

**Affiliations:** 1 Department of Physical Therapy, Federal University of Minas Gerais (UFMG), Av. Antônio Carlos 6627, EEFFTO, Pampulha, Belo Horizonte, Minas Gerais Brazil; 2 Faculty of Medicine, Federal University of Minas Gerais (UFMG), Belo Horizonte, Minas Gerais Brazil; 3 University of São Paulo (USP), Ribeirão Preto, São Paulo, Brazil; 4 Campinas State University (UNICAMP), Campinas, São Paulo, Brazil; 5 Rio de Janeiro State University (UERJ), Rio de Janeiro, Rio de Janeiro Brazil

**Keywords:** Frailty, Older adults, Social, Psychological

## Abstract

**Background:**

Frailty is a predictor of negative health outcomes in older adults. The physical frailty phenotype is an often used form for its operationalization. Some authors have pointed out limitations regarding the unidimensionality of the physical phenotype, introducing other dimensions in the approach to frailty. This study aimed to create a multidimensional model to evaluate frailty in older Brazilian adults and to compare the dimensions of the model created among the categories of the physical frailty phenotype.

**Methods:**

A cross-sectional study was conducted using data from 3569 participants (73.7 ± 6.6 years) from a multicenter and multidisciplinary survey (FIBRA-BR). A three-dimensional model was developed: physical dimension (poor self-rated health, vision impairment, hearing impairment, urinary incontinence, fecal incontinence, and sleeping disorder), social dimension (living alone, not having someone who could help when needed, not visiting others, and not receiving visitors), and psychological dimension (depressive symptoms, concern about falls, feelings of sadness, and memory problems). The five criteria of the phenotype created by Fried and colleagues were used to evaluate the physical frailty phenotype. The proposed multidimensional frailty model was analyzed using factorial analysis. Pearson’s chi-square test was used to analyze the associations between each variable of the multidimensional frailty model and the physical phenotype categories. Analysis of variance compared the multidimensional dimensions scores among the three categories of the physical frailty phenotype.

**Results:**

The factorial analysis confirmed a model with three factors, composed of 12 variables, which explained 38.6% of the variability of the model data. The self-rated health variable was transferred to the psychological dimension and living alone variable to the physical dimension. The vision impairment and hearing impairment variables were dropped from the physical dimension. The variables significantly associated with the physical phenotype were self-rated health, urinary incontinence, visiting others, receiving visitors, depressive symptoms, concern about falls, feelings of sadness, and memory problems. A statistically significant difference in mean scores for physical, social, and psychological dimensions among three physical phenotype categories was observed (*p* < 0.001).

**Conclusions:**

These results confirm the applicability of our frailty model and suggest the need for a multidimensional approach to providing appropriate and comprehensive care for older adults.

## Background

Frailty is a condition that has broadly been investigated in geriatrics and gerontology fields in the last decades. Although there are important conceptual variations, frailty has been commonly defined as reduced physiological reserves and diminished resistance capacities of the human body in response to stressful internal or external situations [1]. Based on that definition, Fried and colleagues (2001) proposed a phenotype for frailty using the physical criteria of the Cardiovascular Health Study [2]. According to this phenotype, individuals with three or more of the following criteria are considered frail: unintentional weight loss, self-reported exhaustion, low physical activity level, muscle weakness, and slow walking speed. Those with one or two criteria are considered pre-frail. Although the physical phenotype has standardized the measurement, there is still great variability in the results across studies [3].

On the other hand, other researchers have adopted a multidimensional approach to evaluate frailty. Some studies have demonstrated the importance of considering both psychological and social dimensions beyond physical criteria [4–6]. A group of Dutch and North American experts developed an integrative definition of frailty as a dynamic state that affects the individual in one or more functioning domains (physical, psychological, and social), which increases the risk of adverse health outcomes [4]. Notably, frailty has been found to be a more robust indicator than chronological age for some negative outcomes related to aging, such as institutionalization, falls, hospitalization, mortality [2, 7], and low quality of life [8], and it has been also considered to be a state that precedes functional disability [7].

There are several multidimensional instruments available for assessing frailty in the literature, such as the Frailty Index [9], Tilburg Frailty Indicator [8, 10], Groningen Frailty Indicator [11], Comprehensive Frailty Assessment Instrument (CFAI) [11, 12], and Edmonton Frailty Scale [13]. The Frailty Index or Accumulated Deficit Index developed by Rockwood and Mitnitski was the first proposed instrument that incorporated the multidimensional nature in the operational definition of frailty [10]. Afterward, the Tilburg Frailty Indicator was proposed to identify the three functioning domains (social, psychological, and physical) [4]. Recently, the International Clinical Practice Guidelines for Physical Frailty indicated the physical phenotype as a good instrument for classifying the frailty stage but pointed out the need to complement information from other human functioning domains, including social, psychological, and physical parameters [14].

Corroborating this discussion, systematic review on the prevalence of frailty in community-dwelling older adults based on 21 cohorts involving 61,500 participants found that the reported prevalence rates differed substantially between the included studies, ranging from 4 to 59.1%. According to the authors, this finding is strongly related to the diversity of frailty conceptualizations. Using physical criteria, the prevalence ranged from 4 to 17%. On the other hand, studies that used broad definitions of frailty incorporating physical, psychological, and/or social dimensions of frailty found prevalence rates from 4.2 to 59.1% [15]. Similarly, a recent systematic review on the prevalence of frailty in Latin American and Caribbean countries showed a large variation of prevalence, with rates ranging from 7.7 to 42.6% [16]. In Brazil, a recent study comparing the prevalence of frailty using the physical phenotype and the Tilburg Frailty Indicator among older users of primary health care found frailty prevalence of 23.5 and 35.8%, respectively [17].

Although frailty is a dynamic and multidimensional condition, most studies usually use physical criteria to evaluate frailty [11]. On the other hand, an approach by integrating health, functioning, social involvement, and well-being [9, 18] is appropriate and quite important in clinical settings. Nevertheless, few previous studies have taken a multidimensional approach to frailty in Brazil [17, 19, 20].

The Frailty in Brazilian Older People Study (FIBRA-BR) analyzed community-dwelling older adults using the physical phenotype as a theoretical framework, which improved the understanding of frailty in Brazil. However, a multidimensional approach could broaden the knowledge by including other indicators related to aging and thereby initiate new areas of research. Therefore, the objectives of the present study were to create a three-dimensional model to assess frailty in older Brazilian adults based on the Tilburg Frailty Indicator [4] and variables available in the FIBRA-BR study database and to compare the dimensions of the model created between the categories of the physical frailty phenotype.

## Methods

### Study design and participants

This cross-sectional study used data from the Frailty in Brazilian Older People Study (FIBRA-BR), a multidisciplinary and multicenter survey about frailty in a sample of 6762 Brazilian community-dwelling older adults conducted between 2009 and 2010. Four public universities were responsible for training, data collection, and data storage in four groups of Brazilian cities. The 15 cities were chosen based on convenience. Participants in each city were selected using probabilistic sampling methods and stratified by sex and age. Methodological details of the sampling are available elsewhere [21].

Inclusion criteria for the FIBRA-BR study were as follows: (1) living in the community, (2) age 65 years or older, (3) both sexes, and (4) ability to ambulate with or without assistance or walking-devices. The exclusion criteria were: (1) cognitive impairment defined as a score less than 17 on the Mini-Mental State Examination [22], (2) motor impairments and aphasia due to stroke, (3) diagnosis of severe or unstable Parkinson’s disease, (4) terminal illness, (5) current cancer treatment, (6) temporary or permanent use of a wheelchair, and (7) being bedridden. In addition, participants with incomplete data on the multidimensional frailty dimensions were excluded from the analytical sample. This study was conducted in strict adherence with the principles of the Declaration of Helsinki. The research ethics committee of the Federal University of Minas Gerais approved the study protocol (ETIC 187/07). All participants signed an informed consent form in advance of their participation.

### Variables

#### Physical phenotype

In this study, we used the five criteria of the physical phenotype created by Fried and colleagues [2] . The criteria include (1) unintentional weight loss of more than 4.5 kg during the past year or loss of 10% of total body weight; (2) self-reported exhaustion evaluated by two questions from the Center for Epidemiological Studies-Depression Scale (CES-D): “How often in the last week did you feel that everything you did was an effort?” and “How often in the last week did you feel that you could not get going?”. Answering “always” or “most of the time” to one of the questions was considered positive for this criterion; (3) low physical activity level measured by caloric expenditure using the Minnesota Leisure Time Activities Questionnaire, which was translated and adapted into Brazilian Portuguese [23]; (4) weak handgrip strength measured by the JAMAR® dynamometer; and (5) slow walking speed indicated by time spent to walk a distance of 4.6 m at a self-selected pace. Participants were classified as frail if they presented three or more criteria, pre-frail if they presented one or two criteria, and non-frail if they presented none of the criteria [2].

#### Multidimensional frailty model

The multidimensional frailty model proposed by this study comprised physical, social, and psychological dimensions of frailty based on the Tilburg Frailty Indicator [4] and adapted multidimensional frailty models [6, 18]. The dimensions were composed of the variables available in the FIBRA-BR study database in order to represent each dimension of the integrative approach of the Tilburg Frailty Indicator.

#### Physical dimension

The physical criteria comprised the following self-reported variables: hearing impairment, vision impairment, urinary incontinence, fecal incontinence, and sleeping disorder (yes or no). Self-rated health was evaluated by asking “In general, how would you say your health is?”. The response options regular, poor, and very poor indicated poor self-rated health, and excellent, very good, and good indicated good self-rated health.

#### Social dimension

The social dimension included questions about the social support network and social connectedness. The self-reported variables were living alone (yes or no), having someone who could help when needed (yes or no), visiting others (still does or never/not anymore), and receiving visitors (still does or never/not anymore).

#### Psychological dimension

**Fig. 1:**
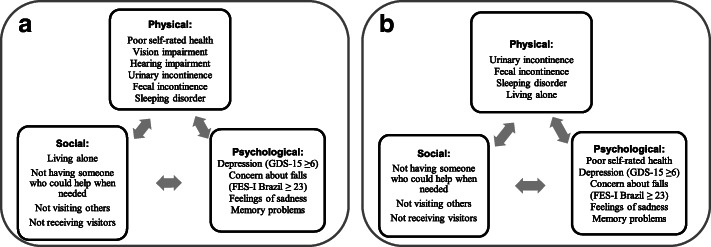
**a** Proposed multidimensional frailty model; **b** Final multidimensional frailty model after factorial analysis: physical dimension (range: 0–4); social dimension (range 0–3); psychological dimension (range 0–5). Source: Adapted from the Tilburg Frailty Indicator (Gobbens et al., 2010). Note: GDS-15 = Geriatric Depression Scale with 15 items; FES-I Brazil = Brazilian version of the Falls Efficacy Scale–International

The psychological dimension of frailty was measured in terms of depressive symptoms evaluated using the Brazilian version of the Geriatric Depression Scale with 15 items (GDS-15) [24], with a score of six or higher suggesting the presence of depressive symptoms [25]. The concern about falls when performing daily activities was evaluated using the Brazilian version of the Falls Efficacy Scale–International (FES-I Brazil) [26], with a score of 23 or higher indicating high concern about falls [27]. Self-reported feelings of sadness during the past 12 months and short-term and long-term memory problems were also used (yes or no). Our multidimensional frailty model is presented in Fig. 1a.

### Statistical analysis

Frequency distributions for categorical variables and measures of central tendency and variability for numerical variables were used for descriptive analyses. First, Pearson’s chi-square test was used to analyze the associations between each variable of the multidimensional frailty model and the physical phenotype categories. The multidimensional frailty model was analyzed using a factorial analysis with the principal component method and varimax rotation in the variables that were used to measure the physical, social, and psychological dimensions. The Kaiser-Meyer-Olkin (KMO) measure and Bartlett’s sphericity test were used to assess the adequacy of the final model. Variables with factorial loadings lower than 0.40 or simultaneously high loadings in two factors were excluded [28]. Subsequently, the scores in each dimension were summed. The score for each dimension corresponded to the sum of the items considered positive, and varied according to the number of items that remained in the factor (dimension) after the factorial analysis (Fig. 1b). No cutoff points were proposed for any dimension or a total score. These composite scores on each dimension were compared among physical phenotype categories (non-frail, pre-frail, and frail) using analysis of variance (ANOVA) with Tukey post hoc test for multiple comparisons. The SPSS 21.0 statistical package was used to perform all the analyses, and the statistical significance level was set at 5%.

## Results

### Participants’ characteristics

Of the 6762 older adults enrolled in the FIBRA-BR study, 3569 participants (56%) had data on all relevant items used in this study and therefore composed our analytical sample. Their mean age was 73.7 (± 6.6) years; 66.3% were female, 48.4% were married or living with a partner, 34.5% were widowed, 6.4% were divorced, and 10.7% were single. Their mean years of schooling were 4.8 (± 4.7) years, and household income was USD 389.3 (± 548.4) per month. Other characteristics of the study participants are presented in Table 1.

**Table 1:** Characteristics of the study participants. The Frailty in Brazilian Older People Study (FIBRA-BR), 2009–2010 (*N* = 3569)

	Participants (N = 3569)
Variables	***n*** (%)
Sex (women)	2367 (66.3)
Age (years), mean (SD)	73.7 (6.6)
Schooling (years), mean (SD)	4.8 ± 4.7
Household income (USD), mean (SD)	389.3 ± 548.4
Married or living with partner	1726 (48.4)
Widow/widower	1231 (34.5)
Divorced	229 (6.4)
Single	380 (10.7)
Self-rated health (poor)	1773 (49.8)
Hearing impairment (yes)	930 (26.1)
Vision impairment (yes)	3109 (87.1)
Urinary incontinence (yes)	821 (23.1)
Fecal incontinence (yes)	182 (5.1)
Sleeping disorder (yes)	1598 (45.2)
Living alone (yes)	404 (12.7)
Having someone who could help when needed (no)	376 (10.7)
Visiting others (never/not anymore)	999 (28.1)
Receiving visitors (never/not anymore)	237 (6.7)
Depressive symptoms (GDS-15 score > 6)	2621 (73.6)
Concern about falls (FES-I Brazil score ≥ 23)	1984 (56.0)
Feelings of sadness (yes)	1613 (45.4)
Memory problems (yes)	1855 (52.4)

*n* number, *SD* standard deviation, *USD* American dollar

Of the 3569 participants of the present study, 68% (2441) had data on all five physical frailty criteria. Of these 2441 participants, 993 (40.7%) were non-frail, 1247 (51.1%) were pre-frail, and 201 (8.2%) were frail. The frequency distribution of the physical frailty criteria for these older adults was muscle weakness (28%), slow walking speed (27%), low physical activity level (20%), self-reported exhaustion (20%), and unintentional weight loss (17%).

### Multidimensional frailty model

Table 2 presents the associations between physical phenotype and the variables of the physical dimension of the proposed multidimensional frailty model. There were significant associations between self-rated health and the physical phenotype (*p* < 0.001) and between urinary incontinence and the physical phenotype (*p* < 0.001). The proportions of poor self-rated health and urinary incontinence significantly increased as the frailty level in the physical phenotype increased.

**Table 2:** Associations between the variables of the physical dimension of the proposed multidimensional frailty model and the physical phenotype categories. The Frailty in Brazilian Older People Study (FIBRA-BR), 2009–2010

Physical dimension	Physical phenotype categories			
	Non-frail ***n*** (%)	Pre-frail ***n*** (%)	Frail ***n*** (%)	***p***-value
Self-rated health				
Good	616 (62.0)	596 (47.9)	76 (37.8)	0.001
Poor	377 (38.0)	649 (52.1)	125 (62.2)	
Total	993	1245	201	
Hearing impairment				
No	762 (76.7)	916 (73.5)	153 (76.1)	0.190
Yes	231 (23.3)	331 (26.5)	48 (23.9)	
Total	993	1247	201	
Vision impairment				
No	128 (12.9)	153 (12.3)	24 (11.9)	0.880
Yes	865 (87.1)	1094 (87.7)	177 (88.1)	
Total	993	1247	201	
Urinary incontinence				
No	828 (83.4)	944 (75.8)	133 (66.2)	< 0.001
Yes	165 (16.6)	302 (24.2)	68 (33.8)	
Total	993	1246	201	
Fecal incontinence				
No	961 (96.8)	1184 (95.0)	191 (95.0)	0.109
Yes	32 (3.2)	62 (5.0)	10 (5.0)	
Total	993	1246	201	
Sleeping disorder				
No	548 (55.7)	689 (55.5)	106 (52.7)	0.735
Yes	436 (44.3)	552 (44.5)	95 (47.3)	
Total	984	1241	201	

*n* number

Regarding social dimension, there were significant associations between the physical phenotype and both visiting others (*p* < 0.001) and receiving visitors (*p* = 0.001). The proportion of participants who did not visit others or receive visitors increased as the frailty level in the physical phenotype increased (Table 3).

**Table 3:** Associations between the variables of the social dimension of the proposed multidimensional frailty model and the physical phenotype categories. The Frailty in Brazilian Older People Study (FIBRA-BR), 2009–2010

Social dimension	Physical phenotype categories			
	Non-frail ***n*** (%)	Pre-frail ***n*** (%)	Frail ***n*** (%)	***p***-value
Living alone				
No	700 (88.9)	948 (86.2)	160 (86.0)	0.182
Yes	87 (11.1)	152 (13.8)	26 (14.0)	
Total	787	1100	186	
Having someone who could help when needed				
Yes	880 (89.3)	1082 (87.9)	181 (90.0)	0.460
No	105 (10.7)	149 (12.1)	20 (10.0)	
Total	985	1231	201	
Visiting others				
Still does	796 (80.2)	917 (73.5)	112 (55.7)	< 0.001
Never/not anymore	197 (19.8)	330 (26.5)	89 (44.3)	
Total	993	1247	201	
Receiving visitors				
Still does	948 (95.5)	1169 (93.7)	178 (88.6)	0.001
Never/not anymore	45 (4.5)	78 (6.3)	23 (11.4)	
Total	993	1247	201	

*n* number

Table 4 shows the associations between physical phenotype and the variables of the psychological dimension of the proposed multidimensional frailty model. All variables were significantly associated with the physical phenotype (*p* ≤ 0.001) The proportions of older adults with depressive symptoms, high concern about falls, and feelings of sadness increased as the frailty level in the physical phenotype increased. On the other hand, those who were pre-frail or frail were equally likely to report memory problems (55%).

**Table 4:** Associations between the variables of the psychological dimension of the proposed multidimensional frailty model and the physical phenotype categories. The Frailty in Brazilian Older People Study (FIBRA-BR), 2009–2010

Psychological dimension	Physical phenotype categories			
	Non-frail ***n*** (%)	Pre-frail ***n*** (%)	Frail ***n*** (%)	***p***-value
Depressive symptoms (GDS-15)				
Less than 6	417 (42.0)	341 (27.3)	47 (23.4)	0.001
6 or higher	576 (58.0)	906 (72.7)	154 (76.6)	
Total	993	1247	201	
Concern about falls (FES-I Brazil)				
Less than 23	597 (60.4)	537 (43.3)	51 (25.8)	< 0.001
23 or higher	392 (39.6)	704 (56.7)	147 (74.2)	
Total	989	1241	198	
Feelings of sadness				
No	677 (68.2)	656 (52.6)	97 (48.3)	< 0.001
Yes	315 (31.8)	591 (47.4)	104 (51.7)	
Total	992	1247	201	
Memory problems				
No	558 (56.3)	559 (45.0)	90 (45.2)	0.001
Yes	434 (43.8)	682 (55.0)	109 (54.8)	
Total	992	1241	199	

*n* number, *GDS-15* geriatric depression scale with 15 items, *FES-I* falls efficacy scale-international

The results of the factorial analysis revealed a three-factor solution comprised of 12 variables. The final model was highly suitable based on KMO and Bartlett’s sphericity test statistics (Table 5). The number of latent variables that remained in the study after the factorial analysis explained 38.6% of the variability of the model data. The vision impairment and hearing impairment variables were dropped from the physical dimension because the factorial loadings were lower than 0.40. The factorial analysis results were similar to the proposed multidimensional frailty model, except regarding self-rated health (physical dimension) and living alone (social dimension) (Fig. 1). The self-rated health variable was transferred after factorial analysis to the psychological dimension and the living alone variable to the physical dimension.

**Table 5:** Factorial analysis results of 12 variables comprising the three dimensions of the multidimensional frailty model. The Frailty in Brazilian Older People Study (FIBRA-BR), 2009–2010

Variables	Factor 1 Physical	Factor 2 Social	Factor 3 Psychological
Self-rated health			0.563
Urinary incontinence	0.459		
Fecal incontinence	0.471		
Sleeping disorder	0.512		
Living alone	0.512		
Having someone who could help when needed		0.429	
Visiting others		0.691	
Receiving visitors		0.701	
Depressive symptoms			0.426
Concern about falls			0.481
Feelings of sadness			0.579
Memory problems			0.568
Kaiser-Meyer-Olkin (KMO) measure	0.782		
Bartlett’s sphericity test	< 0.001		
Variance explained	38.6%		

### Comparisons between multidimenstional frailty model and physical phenotype

The comparisons of the mean scores of three dimensions of the multidimensional frailty model (obtained after factorial analysis) among the physical phenotype categories (non-frail, pre-frail, and frail) are shown in Table 6. The ANOVA results showed a statistically significant difference in mean scores for physical, social, and psychological dimensions among three physical phenotype categories (*p* < 0.001). For all dimensions, the mean score increased as the frailty level in the physical phenotype increased. The Tukey post hoc test revealed that there was a significant difference in mean scores for the physical dimension between non-frail and pre-frail (*p* = 0.008) and between non-frail and frail (*p* = 0.002), but not between pre-frail and frail (*p* = 0.201). Moreover, there was a significant difference in mean scores for social and psychological dimensions between non-frail and pre-frail, non-frail and frail, and pre-frail and frail (*p ≤* 0.001).

**Table 6:** Comparison of the dimensions scores of the multidimensional frailty model among the physical phenotype categories. The Frailty in Brazilian Older People Study (FIBRA-BR), 2009–2010

Physical phenotype	***n***	Mean	Standard deviation	95% CI for mean	Min	Max	***p***-value
*Factor 1: Physical dimension*							
Non-frail	780	0.76	0.74	0.71–0.82	0.00	4.00	< 0.001
Pre-frail	1094	0.87	0.79	0.83–0.92	0.00	4.00	
Frail	186	0.98	0.85	0.86–1.10	0.00	4.00	
*Factor 2: Social Dimension*							
Non-frail	985	0.35	0.59	0.31–0.39	0.00	3.00	< 0.001
Pre-frail	1231	0.45	0.67	0.41–0.49	0.00	3.00	
Frail	201	0.66	0.74	0.55–0.76	0.00	3.00	
*Factor 3: Psychological Dimension*							
Non-frail	987	2.11	1.33	2.02–2.19	0.00	5.00	< 0.001
Pre-frail	1233	2.84	1.38	2.77–2.92	0.00	5.00	
Frail	196	3.22	1.25	3.05–3.40	0.00	5.00	

*n* number, *CI* confidence interval, *Min* minimum value, *Max* maximum value

## Discussion

The purpose of this study was to explore the frailty data in a model composed of three dimensions (physical, social, and psychological) and comparing these dimensions among the frailty categories of the physical phenotype proposed by Fried and colleagues [2] using a large sample of older Brazilian adults. Our final model was composed of the following variables: urinary incontinence, fecal incontinence, sleeping disorder, and living alone (physical dimension); not having someone who could help when needed, not visiting others, and not receiving visitors (social dimension); poor self-rated health, depressive symptoms, concern about falls, feelings of sadness, and memory problems (psychological dimension). In addition, we found that the three dimensions of our multidimensional model are mostly capable to discriminate among non-frail, pre-frail, and frail older adults. Specifically, we observed that frailty scores in the three dimensions increased as the frailty level in the physical phenotype increased. Also, we observed that self-rated health, urinary incontinence, visiting others, receiving visitors, depressive symptoms, concern about falls, feelings of sadness, and memory problems were significantly associated with the physical phenotype.

Our findings suggest the value of considering other criteria, such as social and psychological in addition to physical criteria in studies on frailty. The multiple comparisons of dimensions scores of the multidimensional frailty model among the physical phenotype categories (non-frail, pre-frail, and frail) revealed differences in all dimensions, with one exception. We found that there was not a statistically significant difference in the physical dimension score between pre-frail and frail older adults. This result demonstrates that it is difficult to distinguish between these two physical phenotype categories categorized by the presence of one to two or by three or more frailty criteria. It also reinforces the previous findings that the transition between pre-frailty and frailty is very common [29, 30].

Many studies have demonstrated a need for a holistic perspective in the management of frail older adults [8]. These studies also showed that several frail older adults change their categories when the classification criteria changed from a physical to a multidimensional approach and that this creates problems for providing appropriate care and delays the diagnosis of frailty [6, 31]. Thus, using the variables of the dimensions of our model might help to identify more precisely and early the older adults’ frailty.

Regarding individual variables of physical dimension defined after factorial analysis (urinary incontinence, fecal incontinence, sleeping disorder, and living alone), only urinary incontinence was associated with physical phenotype and was more prevalent as the frailty level in the physical phenotype increased. These results suggest a dose-response relationship and indicate the importance of identifying and proposing preventive actions to help control urinary incontinence. Notably, the low percentage of self-reported urinary incontinence in our study (23.1%) might be explained by the older adults’ misinterpretation who do not consider any involuntary urine loss as urinary incontinence. In addition, older adults tend to deny that they have this health problem due to embarrassment [32].

In disagreement with the model initially proposed from the literature review (Fig. 1a), in the present study, the variable living alone was placed in the physical domain after factorial analysis (Fig. 1b). Moreover, a low percentage of participants reported living alone (about 13%), and this variable was not significantly associated with the physical frailty phenotype. Unlike the present study, Op Het Veld et al. (2015) showed that frail older adults according to physical phenotype were more likely to live alone than those in the other two categories. This divergence between studies might somewhat be explained by Brazilian family arrangements, which are characterized by financial interdependence in families [33]. Thus, regardless of the frailty level, few older adults live alone in Brazil.

Previous studies showed that the living alone variable was related to the social network and social connectedness [18, 34, 35]. On the other hand, literature also reports older adults who live alone might have physical problems that limit their mobility and keep them housebound, which tends to exacerbate their physical problems [36]. Further, living alone might be related to personal strategies and everyday lifestyle adaptations intended to compensate for functional losses, and it might indicate functional decline caused by loss of physiological reserves, decreased physical fitness, and consequent physical frailty [37]. Thus, living alone is also related to the physical dimension, as we found in the present study.

The variables visiting others, receiving visitors, and having someone who could help when needed have composed the social dimension of our multidimensional frailty model. The network of social support (making and receiving visits) decreased as the frailty level in the physical phenotype increased. These results corroborate other studies showing the association between physical frailty criteria and the size of social support network [1, 18, 36]. Unlike the present study, other authors found no difference between the social dimension and frailty categories [10, 12, 18]. For example, Op Het Veld et al. (2015) found no difference in the social support network among the three categories of physical phenotype, although frail older adults became more family dependent as they lose other types of social support. These studies evaluated the social support network as a family dependent, locally integrated, neighborhood-focused and private [18], loneliness [12], and having someone close to the older adults [10], whereas the present study evaluated as the self-report of visiting and receiving visits.

The community-dwelling older Brazilian adults with low income and without the support of public policies present a limited social support network, besides the family [25]. The older Brazilian adults habitually visit others as an important social activity, and physical frailty decreases their ability to do so. Older adults with relatively large social networks apparently have more opportunities to go out to socialize, interact with others, and control the adverse effects of frailty [30]. A previous study showed that older adults with weak or small social support networks were relatively depressed and had limited regular activities [33]. A Dutch study found that the loss of relationships, social support (visits), and other aspects of the social dimension of the frailty integrated model were associated with low quality of life [38]. Therefore, promoting social activities and involvement might help to prevent social vulnerability and avoid its negative consequences [39].

Statistically significant associations were found between all variables of the psychological dimension and the physical phenotype. Thus, poor self-rated health, depressive symptoms, concern about falls, feelings of sadness, and memory problems could complement the physical phenotype proposed by Fried and colleagues [2]. These results might help to guide programs to protect older adults and reduce psychological frailty and its consequences. In line with our findings, previous studies showed a higher proportion of participants with depressive symptoms evaluated by the GDS-15 [40] and high concern about falls [41] measured with the FES-I among frail older adults compared to non-frail older adults.

Self-rated health is an indicator of health in aging, regardless of the frailty level [18]. The integrated frailty model proposed by Gobbens and colleagues (2010) includes self-rated health in the physical dimension [4]. However, we found that self-rated health was a better fit in the psychological than the physical dimension. This result might reflect subjective well-being that includes individuals’ considerations of non-physical health aspects, such as life satisfaction or general happiness. In addition, self-rated health might be influenced by feelings about functioning and/or autonomy rather than disease and illness [42]. From this perspective, health and well-being could be a psychological dimension, as our study found.

This study has some limitations. First, a great number of participants enrolled in the FIBRA-BR study were excluded from the analyses due to missing data, which could interfere in the inference ability of our study. Second, other variables such as loneliness, network size, contact frequency, and emotional support were not investigated in the FIBRA-BR study. Therefore, future studies should include these variables to provide further insight into multidimensional approaches for frailty in low-and-middle-income countries, such as Brazil. Lastly, due to the eligibility criteria of the FIBRA-BR study, our results cannot be generalized for older adults with greater functional or cognitive decline. On the other hand, the current study presents some strengths that should be highlighted. This study was conducted with a large sample of older adults of both sexes from various Brazilian cities with different human development indexes, which enhances the generalization of our findings. The variables included in our model are easily obtained in clinical practice. Thus, our multidimensional frailty model has the potential to be used in this setting. Lastly, the adoption of standardized procedures, extensive training of the field personal, and face-to-face interviews at older adults’ homes contributed to the high quality of data collected.

## Conclusions

This study confirmed the adequacy of a proposed multidimensional frailty model, which moderately explained the variance of the variables selected to evaluate frailty. The self-rated health, urinary incontinence, visiting others, receiving visitors, depressive symptoms, concern about falls, feelings of sadness, and memory problems were significantly associated with the physical phenotype. Furthermore, we observed significant differences in mean scores of physical, social, and psychological dimensions among the physical phenotype categories, indicating that our multidimensional frailty model is able to discriminate among non-frail, pre-frail, and frail older adults according to the classification proposed by Fried and colleagues [2]. Our results suggest the need for a multidimensional approach to provide complete care for older adults at different frailty levels and to progress further in research on frailty in Brazil.

## Data Availability

The datasets generated used and/or analysed during the current study available from the corresponding author on reasonable request.

## Abbreviations

FES-I: Falls efficacy scale-international
FIBRA-BR: Fraily in Brazilian Oder People Study
GDS-15: Geriatric depression scale with 15 items
KMO: Kaiser-Meyer-Olkin

## References

[1] Hoogendijk EO, Suanet B, Dent E, Deeg DJ, Aartsen MJ (2016). Adverse effects of frailty on social functioning in older adults: results from the longitudinal aging study Amsterdam. Maturitas..

[2] Fried LP, Tangen CM, Walston J, Newman AB, Hirsch C, Gottdiener J, Seeman T, Tracy R, Kop WJ, Burke G, McBurnie MA (2001). Cardiovascular health study collaborative research group. Frailty in older adults: evidence for a phenotype. J. Gerontol A Biol Med Sci.

[3] Theou O, Cann L, Blodgett J, Wallace LMK, Brothers TD, Rockwood K (2015). Modifications to the frailty phenotype criteria: systematic ther current literature and investigation of 262 frailty phenotype the survey of health, ageing, and retirement in Europe. Ageing Res Rev.

[4] Gobbens RJ, Luijky KG, Wijnen-Sponselee MT, Schols JM (2010). Toward a conceptual definition of frail community dwelling older people. Nurs Outlook.

[5] Schuurmans H, Steverink N, Lindenberg S, Frieswijk N, Slaets JPJ (2004). Old or frail: what tells us more?. J Gerontol A Biol Med Sci.

[6] van Oostrom SH, van Der ADL, Rietman ML, Picavet HSJ, Lette M, Verschuren WMM, Bruin SR, Spijkerman AMW (2017). A four-domain approach of frailty explored in the Doetinchem cohort study. BMC Geriatr.

[7] Fried LP, Ferrucci L, Darer J, Williamson JD, Anderson G (2004). Untangling the concepts of disability, frailty, and comorbidity: Implications for improved targeting and care. J Gerontol A Biol Med Sci.

[8] Gobbens RJ, van Assen MA (2014). The prediction of ADL and IADL disability using six physical indicators of frailty: a longitudinal study in the Netherlands. Curr Gerontol Geriatr Res.

[9] Rockwood K, Mitnitski A (2007). Frailty in relation to the accumulation of deficit. J Gerontol A Biol Med Sci.

[10] Roppolo M, Mulasso A, Mossso CO, Rabaglietti E (2015). A comparison between uni- and multidimensional frailty measures: prevalence, functional status, and relationships with disability. Clin Interv Aging.

[11] Dent E, Kowal P, Hoogendikj EO (2016). Frailty measurement in research and clinical: a review. Eur J Intern Med.

[12] Van der Elst MCJ, Schoenmakers, B, Op het Veld LPM, De Roeck, EE, Van der Vorst A, Kempen GIJM, De Witte N, Lepeleire JD, Schols, JMG A Concordances and differences between a unidimensional and multidimensional assessment of frailty: a cross-sectional study BMC Geriatrics 2019; 19: 346. doi: 10.1186/s12877-019-1369-7. 10.1186/s12877-019-1369-7 PMC6902576 31822285

[13] Rolfson DB, Majumdar SR, Ross T, Tahir A, Rockwood K. Validity and reliability of the Edmonton frail scale. Ageing. 2006. 10.1093/ageing/af1023. 10.1093/ageing/afl041 PMC5955195 16757522

[14] Dent E, Morley JE, Cruz-Jentoft AJ, Woodhouse L, Rodríguez-Manãs L, Fried LP, Woo J, Aprahamaina I, Sanford A, Lundy J (2019). Physical frailty: ICFSR International clinical practice guidelines for identification and management. J Nutr Health Aging.

[15] Collard RM, Boter H, Schoevers RA, Oude Voshaar RC (2012). Prevalence of frailty in community-dwelling older persons: a systematic review. J Am Geriatr Soc.

[16] Da Mata FAF, Pereira PPS, Andrade KRC, Figueiredo ACMG, Silva MT, Pereira MG (2016). Prevalence of frailty in Latin America and the Caribbean: A systematic review andmeta-analysis. Plos One.

[17] Santiago LM, Gobbens RJJ, Mattos IE, Ferreira DB (2019). A comparison between physical and biopsychosocial measures of frailty: prevalence and associated factors in Brazilian older adults. Arch Gerontol Geriatr.

[18] Op het Veld LP, van Rossum E, Kempen GIJM, de Vet HCW, Hajema KJ, Beurskens AJHM. Fried phenotype of frailty: Cross-sectional comparison of three frailty stages on various health domains. BMC Geriatr. 2015; 15:77. doi: 10.1186/s12877-015-0078-0. 10.1186/s12877-015-0078-0 PMC4496916 26155837

[19] Malini MF, Lourenço RA, Lopes CS (2016). Prevalence of fear of falling in older adults, and its associations with clinical, functional and psychosocial factors: the frailty in Brazilian older people-Rio de Janeiro study. Geriatr Gerontol Int.

[20] Pereira AA, Borim FSA, Neri AL (2017). Absence of association between frailty index and survival in elderly Brazilians: the FIBRA study. Cad. Saúde Pública..

[21] Neri AL, Yassuda MS, Araújo LF, Eulálio MC, Cabral BE, Siqueira MEC (2013). Metodology and social, demographic, cognitive, and frailty profiles of community-dwelling elderly from seven Brazilian cities: the FIBRA study. Cad Saúde Pública.

[22] Brucky SMD, Nitrini R, Caramelli P, Bertolucci PHF, Okamoto IH (2003). Suggestions for utilization of the mini-mental state examination in Brazil. Arq Neuropsiquiatr.

[23] Lustosa LP, Pereira DS, Dias RC, Britto RR, Parentoni NA, Pereira LSM (2011). Translation and cultural adaptation of the Minnesota leisure time activities questionnaire in community-dwelling older people. Geriatr Gerontol.

[24] Almeida OP, Almeida SA (1999). Reliability of the Brazilian version of the geriatric depression scale (GDS) short form. Arq Neuropsiquiatr.

[25] Herrmann N, Mittmann N, Silver IL, Shulman KI, Busto UA, Shear NH (1996). A validation study of the geriatric depression scale short form. Int J Geriatr Psychiatry.

[26] Camargos FF, Dias RC, Dias JM, Freire MT (2010). Cross-cultural adaptation and evaluation of the psychometric properties of the falls efficacy scale-international among elderly Brazilians. Braz J Phys Ther.

[27] Delbaere K, Close JC, Mikolaizak AS, Sachdev PS, Brodaty H, Lord SR (2010). The falls Efficay scale international (FES-I). A comprehensive longitudinal validation study. Age Ageing.

[28] Hair JF, Anderson RE, Tatham RL, Black WC (1995). Multivariate data analysis with readings.

[29] Gill TM, Gahbauer EA, Allore HG, Han L (2016). Transitions between frailty states among community-living older persons. Arch Intern Med.

[30] Faria GS, Ribeiro TMS, Vieira RA, Silva SLA, Dias RC (2016). Transition between frailty leves in elderly persons from Belo Horizonte, Minas Gerais, Brazil. Rev Bras Geriatr Gerontol.

[31] van Assen MA, Pallast E, Fakiri FE, Gobbens RJ. Measuring frailty in Dutch community-dwelling older people: Reference values of the Tilburg Frailty Indicator (TFI). Arch Gerontol Geriatr. 2016; (67),120–9. doi: 10.1016/j.archger.2016.07.005. 10.1016/j.archger.2016.07.005 27498172

[32] Buckley BS, Lapitan MC. Prevalence of urinary incontinence in men, women, and children-current evidence: Findings of the fourth international consultation on incontinence. Urology. 20010; 76(2): 265–70. doi: 10.1016/j.urology.2009.11.078. 10.1016/j.urology.2009.11.078 20541241

[33] Melo NCV, Teixeira KMD, Barbos TL, Montoya AJA, Silveir MB (2016). Household arrangements of elderly persons in Brazil: Analyses based on the national household survey sample (2009). Rev Bras Geriatr Gerontol.

[34] Makizako H, Shimada H, Doi T, Tsutsumimoto K, Hotta R, Nakakubo S, Makino K, Lee S (2018). Social frailty leads to the development of physical frailty among physically non-frail adults: A four-year follow-up longitudinal cohort study. Int J Environ Res Public Health.

[35] Tsutsumimoto K, Doi T, Makizako H, Hotta R, Nakakubo S, Kim M, et al. Social frailty has a stronger impact on the onset of depressive symptoms than physical frailty or cognitive impairment: A 4-year follow-up longitudinal cohort study. JAMDA. 19(6):504–10. 10.1016/j.jamda.2018.02.008. 10.1016/j.jamda.2018.02.008 29703687

[36] Sakurai R, Kawai H, Suzuki H, Kim H, Watanabe Y, Hirano H, Ihara K, Obuchi S, Fujiwara Y (2019). Poor social network, not living alone, is associated with incidence of adverse health outcomes in older adults. JAMDA..

[37] Meng LD, Liu YC, Feng X, Zhai YX, Liu K (2018). The mediating role of depression on the relationship between housebound status and cognitive function among the elderly in rural communities: a cross-sectional study. Arch Gerontol Geriatr.

[38] Levasseur M, Généreux M, Bruneau JF, Vanasse A, Chabot E, Beaulac BMM. Importance of proximity to resources, social support, transportation and neighborhood security for mobility and social participation in older adults: Results from a scoping study. BMC Public Health. 2015;15(503). 10.1186/s12889-015-1824-0. 10.1186/s12889-015-1824-0 PMC4460861 26002342

[39] Renne I, Gobbens RJ (2018). Effects of frailty and chronic diseases on quality of life in Dutch community-dwelling older adults: a cross-sectional study. Clinic Interv Aging.

[40] Vieira RA, Guerra RO, Giacomin KC, Vasconcelos KSS, Andrade ACS, Pereira LSM (2013). Prevalence of frailty and associated factors in community-dwelling elderly in Belo Horizonte, Minas Gerais state, Brazil: data from the FIBRA study. Cad Saúde Pública.

[41] Esbrí-Víctor M, Huedo-Rodenas I, López-Utiel M, Navarro-López JL, Martínez-Reig M, Serra-Rexach JA, et al. Frailty and fear of falling: The FISTAC Study. J Frailty Agind. 6(3):136–40. 10.14283/jfa.2017.19. 10.14283/jfa.2017.19 28721429

[42] Dutra FCMS, Mancini MC, Neves JA, Kirkwood RN, Sampaio RF (2016). Empirical analysis of the international classification of functioning, disability and health (ICF) using structural equation modeling. Braz J Phys Ther..

